# Inhaled nitric oxide in fibrotic and advanced interstitial lung disease: A systematic review and meta-analysis of randomized controlled trials

**DOI:** 10.1371/journal.pone.0351862

**Published:** 2026-06-22

**Authors:** Shun Nakahara, Yusuke Hirao, Bradley Fujiuchi, Brent Matsuda

**Affiliations:** 1 Department of Medicine, John A. Burns School of Medicine, University of Hawaii, Honolulu, Hawaii, United States of America; 2 Pulmonary and Critical Care Division, Queen’s University Medical Group, Honolulu, Hawaii, United States of America; Rutgers Biomedical and Health Sciences, UNITED STATES OF AMERICA

## Abstract

**Purpose:**

Interstitial lung disease (ILD) can lead to pulmonary hypertension (PH), contributing to reduced exercise capacity in patients with ILD. Inhaled nitric oxide (iNO) reduces pulmonary artery pressure and pulmonary vascular resistance and may improve exercise capacity in this population; however, the available evidence remains limited. Therefore, we conducted a systematic review and meta-analysis to compare the efficacy and safety of iNO versus placebo in patients with ILD.

**Methods:**

We systematically searched PubMed, Embase, Cochrane databases, and ClinicalTrials.gov for randomized controlled trials (RCTs) comparing iNO with placebo in patients with ILD. The primary endpoint was 6-minute walk distance (6MWD). Secondary endpoint was moderate-to-vigorous physical activity (MVPA). Safety endpoint was any adverse events. Continuous endpoints were reported using mean differences (MDs) or standardized mean differences (SMDs), and binary endpoints were reported using risk ratios (RRs), all with 95% confidence intervals (CIs).

**Results:**

Four RCTs, including one crossover trial, enrolling 274 patients were analyzed. Of these, 172 received iNO. The median follow-up period was 12 weeks. In the pooled analyses, iNO likely results in little to no difference in 6MWD compared with placebo (MD 1.83m; 95% CI −12.98 to 16.64; p = 0.81), and may result in little to no difference in any adverse events (RR 1.12; 95% CI 0.97 to 1.30; p = 0.12) and MVPA (SMD 0.32; 95% CI −0.93 to 1.56; p = 0.39).

**Conclusion:**

INO did not demonstrate improvements in 6MWD or MVPA. These findings do not support routine use of iNO in this population.

## Introduction

Interstitial lung disease (ILD) represents a heterogeneous group of disorders with considerable diagnostic complexity, in which inflammatory and fibrotic processes frequently coexist, complicating treatment strategies [1]. Currently FDA-approved antifibrotic agents such as nintedanib, pirfenidone, and nerandomilast are the standard treatments for patients with progressive pulmonary fibrosis and idiopathic pulmonary fibrosis (IPF) [2]. In patients with concomitant pulmonary hypertension (PH), inhaled treprostinil may offer benefits in exercise capacity and respiratory symptoms [3]. However, the overall impact of current therapies on exercise tolerance remains limited [4,5]. In this context, inhaled nitric oxide (iNO) has emerged as a potential therapeutic option [6].

Nitric oxide (NO) induces selective pulmonary vasodilation by activating the cyclic guanosine monophosphate pathway in vascular smooth muscle, while being rapidly inactivated by hemoglobin to prevent systemic effects [7]. Through the selective pulmonary effects, iNO may improve ventilation-perfusion matching and alleviate dyspnea by preferentially dilating vessels adjacent to well-ventilated alveoli [8]. Despite this mechanistic rationale, evidence supporting its clinical effectiveness in patients with ILD remains limited. Although iNO is generally considered safe, potential adverse effects at higher doses and prolonged use include methemoglobinemia and rebound pulmonary edema [9–11].

Previous randomized controlled trials (RCTs) have demonstrated increases in moderate-to-vigorous physical activity (MVPA), along with improvements in dyspnea, quality-of-life measures, and oxygenation [6,12]. In contrast, a phase-3 trial in patients with fibrotic ILD found no significant differences in activity level, 6-minute walk distance (6MWD), or patient-reported outcomes; nonetheless, uncertainty persists due to the reduction of the intended sample size caused by slow enrollment, combined with broad confidence intervals observed in a primary outcome [13]. Taken together, the available evidence regarding iNO in ILD remains inconsistent, and limited to small-scale trials. To address these uncertainties, we conducted a systematic review and meta-analysis of RCTs comparing iNO with placebo in patients with ILD. This review sought to determine whether iNO provides clinically meaningful improvements in exercise tolerance outcomes, including functional exercise capacity and daily physical activity, while considering treatment-related adverse events.

## Methods

This systematic review and meta-analysis was performed following the Cochrane Collaboration Handbook for Systematic Review of Interventions and the Preferred Reporting Items for Systematic Reviews and Meta-Analysis (PRISMA) guidelines (S1 Table) [14]. Additionally, our study protocol was registered using the following registration number CRD420251267394 in the International Prospective Register of Systematic Reviews.

### Eligibility criteria

We included studies that met all the following eligibility criteria: (1) RCTs; (2) comparison of iNO with placebo; (3) enrollment of patients with ILD; and (4) reporting of at least one of the clinical outcomes of interest. There were no restrictions on the follow-up duration or language. Citations were excluded if patient populations overlapped with those of other included studies. When the enrollment period was not reported and potential population overlap could not be determined, the corresponding authors were contacted for clarification. Detailed eligibility criteria for each included study are provided in S2 Table.

### Search strategy and data extraction

We systematically searched PubMed, Embase, Cochrane Central Register of Controlled Trials, and ClinicalTrials.gov from inception to December 21, 2025, with the following search terms: “Lung Diseases, Interstitial,” “pulmonary fibrosis,” and “Nitric Oxide,.” The complete search strategy is presented in S3 Table.

References from all included studies, previous systematic reviews, and meta-analyses were manually searched for additional studies. Two authors independently screened and extracted data using dedicated spreadsheets. Any discrepancies were resolved through consultation with a third author.

### Endpoints

Our primary efficacy and safety endpoints were 6MWD and any adverse events, respectively. We also included MVPA as a secondary endpoint. The definitions of each endpoint used in the included studies are provided in S4 Table. We contacted corresponding authors by email to obtain missing outcome data, if necessary.

### Assessment of the risk of bias and quality of evidence

Two authors independently assessed the risk of bias and disagreements were resolved with the senior author. Studies were appraised using the Cochrane Collaboration’s tool for assessing risk of bias in randomized trials (RoB-2), which evaluates five domains: (1) randomization process, (2) deviations from intended protocol, (3) missing outcome data, (4) measurement of the outcome, and (5) selection of the reported result [15]. Additionally, we employed the RoB-2 tool for crossover trials for relevant studies, which incorporates specific assessments for bias arising from period and carryover effects. The quality of evidence was assessed using the Grading of Recommendations Assessment Development and Evaluation (GRADE) approach by two independent reviewers and disagreements were resolved with the third author [16]. We did not assess publication bias due to the limited number of studies included (n < 10), as regression-based methods lack sufficient power to distinguish chance from true asymmetry [17].

### Statistical analysis and sensitivity analyses

We calculated pooled mean differences (MDs) and standardized mean differences (SMDs) for continuous endpoints and risk ratios (RRs) for binary endpoints, accompanied by 95% confidence intervals (CIs) [14]. Missing standard deviations (SDs) were calculated from standard errors (SEs) [14]. For studies reporting only medians, we approximated means and SDs using Wan and Luo’s formulas [18,19]. These statistical transformations were conducted according to the Cochrane guidelines [14]. For outcomes reported using different measurement units, effect sizes were converted to SMDs [14]. The magnitude of these effects was interpreted according to Cohen’s conventions [20], where SMDs of approximately 0.20, 0.50, and 0.80 represent small, moderate, and large effects, respectively. A random-effects model was applied to address between-study heterogeneity due to differences in methodology and patient demographics [14]. Substantial heterogeneity was defined by a Cochrane Q test p-value < 0.10 and I^2^ ≥ 25%. To judge the extent of heterogeneity, 95% prediction Interval (PI) for each component effect was estimated. The PI helps in the clinical interpretation of heterogeneity by estimating what true treatment effects can be expected in future settings. Data synthesis was performed using RStudio (version 4.2.3) with a meta package [18,19]. We performed leave-one-out sensitivity analyses for each outcome to assess the effects of influential studies on the pooled analysis. Studies were sequentially removed one at a time, and the data was reanalyzed to ensure the stability of the pooled effects. Additionally, we performed a sensitivity analysis to assess the impact of skewness introduced by transformations from medians and interquartile ranges to means and SDs, as well as the effect of excluding crossover trials and studies with a high risk of bias.

### Declaration of Generative AI and AI-assisted technologies in the writing process

During the preparation of this work, the authors utilized generative AI (ChatGPT, OpenAI, version 5.2, Gemini) to enhance the clarity and flow of the manuscript. Following the application of this technology, the authors critically reviewed and refined the content to ensure accuracy and take full responsibility for the final integrity of the publication.

### Ethics approval

Institutional Review Board approval was not required for this study, as it is a systematic review and meta-analysis based on previously published data and does not involve direct intervention or interaction with human participants.

## Results

**Fig 1:**
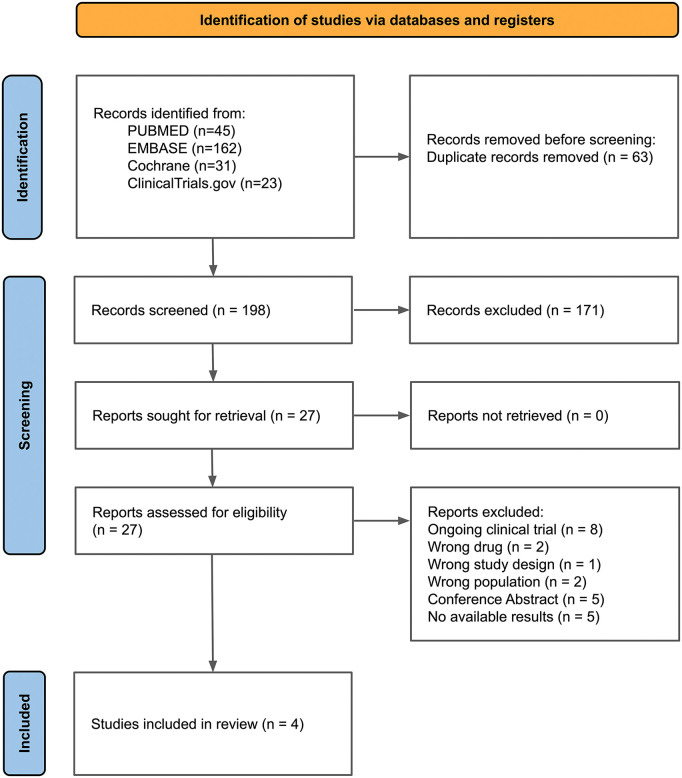
PRISMA Flow Chart.

The initial search yielded 261 results on December 21, 2025. After removing duplicate results and applying the eligibility criteria, 27 records were selected for the full-text review, as detailed in Fig 1. Of these, four RCTs, including one crossover trial, were included in this systematic review and meta-analysis [6,12,13,21]. A total of 274 participants were included, of whom 172 received iNO. A total of 142 (51.8%) patients were classified as having PH, either confirmed by right heart catheterization or based on an intermediate to high echocardiographic probability according to the European Society of Cardiology/European Respiratory Society guidelines.

The age of patients ranged from 63.9 to 70.0 years, and 62.8% were male. Median follow-up period was 12 weeks. INO was administered at doses of 30, 45, or 75 μg/kg of ideal body weight (IBW) per hour, either acutely for 20 minutes or chronically for 8 or 16 weeks. The baseline characteristics of the participants are presented in Table 1 and S5 Table.

**Table 1:** Baseline patient and study characteristics.

Study and year	Type of blinding	Sample size	Population	INO dose rate (μg/kg IBW/h)	INO duration	Follow-up, weeks	Male	Age, years	PH	6MWD, m	DLco predicted,%	FVC predicted,%	Oxygen therapy	IPF
**Freidkin 2024**^**a**^ [21]	Single- blind	44	Advanced ILD	45 or 75	20 minutes once	NA	31 (70.5)	65.5	26 (59.1)	NA	38	57.5	18 (40.9)	25 (56.8)
**King 2022** [12]	Double- blind	44	Fibrotic ILD	45	12 hours/day for 16 weeks	16	25 (56.8)	63.9	27 (61.4)	275.0	35.6	60.0	44 (100.0)	31 (70.1)
**Nathan 2024** [13]	Double-blind	145	Fibrotic ILD	45 or 75	12 hours/day for 16 weeks	16	87 (60.0)	70.0	60 (41.4)	266.8	35.2	59.9	145 (100.0)	79 (54.5)
**Nathan 2020** [6]	Double-blind	41	Fibrotic ILD	30	8 weeks	8	29 (70.7)	67.3	29 (70.7)	284.0	30.5	57.9	41 (100.0)	30 (73.2)

Abbreviations: DLco: diffusing capacity of the lung for carbon monoxide; FVC: forced vital capacity; IBW: ideal body weight; ILD: interstitial lung disease; INO: inhaled nitric oxide; IPF: idiopathic pulmonary fibrosis; NA: not available; PH: pulmonary hypertension; 6MWD: 6-minute walk distance.

a: Crossover design

Categorical data are reported as counts and frequencies (%).

Continuous data are reported as mean or median.

### Pooled analysis of all studies and sensitivity analyses

**Fig 2:**
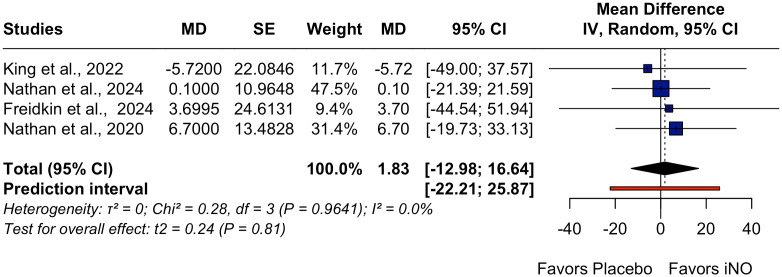
Forest Plot for 6MWD endpoint.

**Fig 3:**
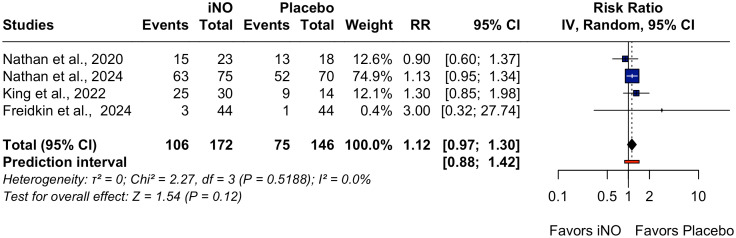
Forest Plot for any adverse events endpoint.

**Fig 4:**
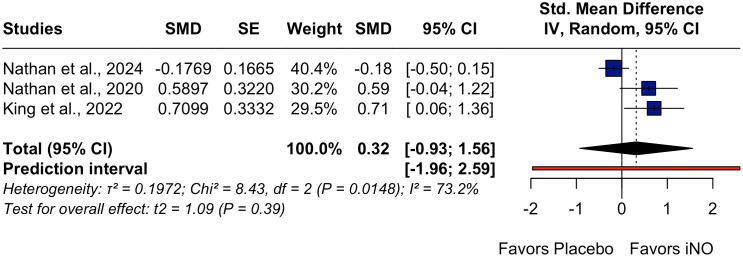
Forest Plot for MVPA endpoint.

We observed no statistically significant differences between the iNO and placebo groups for 6MWD (MD 1.83m; 95% CI −12.98 to 16.64; 95% PI −22.21 to 25.87; I² = 0%; p = 0.81; Fig 2), adverse events (RR 1.12; 95% CI 0.97 to 1.30; 95% PI 0.88 to 1.42; I² = 0%; p = 0.12; Fig 3), or MVPA (SMD 0.32; 95% CI −0.93 to 1.56; 95% PI −1.96 to 2.59; I² = 73%; p = 0.39; Fig 4).

The results of these sensitivity analyses are presented in S4 Figs. For 6MWD, the control group data in one study (Freidkin et al., 2024) appeared skewed following the conversion from median to mean values [21]. However, the leave-one-out analysis demonstrated consistent results after omitting this study. Additionally, the results for both 6MWD and any adverse events remained robust after excluding one crossover trial identified as having a high risk of bias.

The heterogeneity in the MVPA endpoint was primarily driven by one study (Nathan et al., 2024), but excluding this study reduced the I^2^ from 73.9% to 0% [13].

### Risk of bias assessment

Three RCTs were categorized with an overall low risk of bias on the risk of bias-2 (RoB-2) risk assessment tool. One study (Freidkin et al., 2024) was assessed as having a high risk of bias, primarily driven by concerns regarding bias arising from period and carryover effects [21]. Individual study appraisals using the RoB 2 tool are presented in S1 and S2 Figs.

### GRADE assessment

The certainty of evidence was rated as moderate for 6MWD and low for MVPA and any adverse events (S3 Fig). The certainty of evidence for 6MWD and any adverse events was downgraded due to risk of bias, driven by the inclusion of Freidkin et al., 2024 [21]. For MVPA, the certainty of evidence was downgraded for serious inconsistency due to substantial heterogeneity. Imprecision for MVPA was judged to be serious, as the CI was wide and included values consistent with a clinically important benefit. For any adverse events, the certainty of evidence was downgraded for serious imprecision because the CI crossed the line of no effect.

## Discussion

In this systematic review and meta-analysis of four RCTs with 274 patients, iNO was compared with placebo in patients with fibrotic and advanced ILD. Our main results were as follows: (1) there was no difference in 6MWD and MVPA between the iNO and placebo groups and (2) the safety profile of iNO was supported due to similarities in any adverse events endpoint.

ILD is associated with substantial morbidity and mortality, particularly in patients with progressive fibrotic phenotypes and those with PH [22]. INO has been proposed as a potential adjunct to current first-line antifibrotic therapies [6,12]. Previous studies have reported that iNO reduces pulmonary vascular resistance in patients with fibrotic ILD and ILD with PH [23,24]. However, whether these physiological effects translate into clinically meaningful functional improvements remains uncertain.

To address this uncertainty, the outcome of functional exercise capacity has been evaluated in previous studies. The 6MWD is a key prognostic marker in fibrotic ILD, with both baseline values and longitudinal decline strongly associated with survival, particularly in IPF [25,26]. Despite its clinical importance, previous RCTs in patients with advanced and fibrotic ILD have not demonstrated a significant improvement in 6MWD with iNO [13,21]. These findings are consistent with those of our meta-analysis. A previous study also reported that iNO did not significantly affect arterial oxygenation or ventilation–perfusion distribution in patients with ILD [24]. Collectively, these findings indicate that, in patients with advanced and fibrotic ILD, the pathophysiological effects of iNO may be insufficient to result in clinically meaningful improvements in functional exercise capacity [13,21,24].

MVPA, which captures habitual physical activity over several weeks and may better reflect real-world functional status than 6MWD, has shown heterogeneous results across previous RCTs [6,12]. However, in our meta-analysis, no significant difference in MVPA was observed, which is consistent with the findings of a recent phase-3 RCT [13]. In the leave-one-out analysis, exclusion of Nathan et al. (2024) resulted in complete resolution of heterogeneity (I² = 0%), suggesting that this study was the primary contributor to the observed variability [13]. This heterogeneity may be partly explained by differences in baseline characteristics, including the lower prevalence of pulmonary hypertension and older age of the population in Nathan et al. (2024) [13]. These results suggest that, in patients with advanced and fibrotic ILD, iNO may not lead to measurable improvements in this key patient-centered daily physical activity outcome.

The included trials differed in the duration of iNO exposure, which may influence clinical interpretation. Compared with short-term administration, longer-term iNO therapy may offer the potential for more sustained pulmonary hemodynamic effects, including reductions in pulmonary artery pressure and pulmonary vascular resistance [27]. However, prolonged therapy also introduces additional clinical considerations, including device burden, adherence challenges, cost, and the need for careful discontinuation or weaning to avoid potential worsening oxygenation or hemodynamic instability [27,28]. These considerations are important when interpreting the safety and feasibility of iNO therapy in clinical practice.

Regarding safety outcomes, our findings are consistent with those of previous studies, demonstrating no significant difference in any adverse events between iNO and placebo [6,12,13,21]. Although concerns have been raised regarding potential pulmonary tissue injury and methemoglobinemia with higher doses or prolonged use of iNO, our meta-analysis demonstrated iNO may have a favorable safety profile with a mean dose of 45 μg/kg of IBW per hour administered for a mean duration of 16 weeks [29,30]. These results support the tolerability of iNO in patients with advanced and fibrotic ILD.

Our meta-analysis strengthens prior evidence showing no benefit of iNO on 6MWD in advanced and fibrotic ILD and integrates previously variable findings to demonstrate no significant effect on MVPA. Our findings also support the safety of iNO. However, although no functional benefit was observed in this population, studies in other patient populations have reported favorable symptomatic and survival outcomes with iNO therapy [31,32]. In patients with chronic obstructive pulmonary disease, the administration of iNO during exercise reduced dyspnea scores [31]. In critically ill patients with acute right heart failure, the use of iNO was associated with lower mortality among those undergoing heart or lung transplantation [32]. These observations suggest that future studies in ILD may benefit from focusing on quality-of-life–related outcomes.

Our findings should be interpreted in the context of the current therapeutic landscape for ILD. In adult fibrotic ILD, antifibrotic therapy remains the main disease-modifying treatment across age groups, while lung transplantation is considered for selected patients with advanced disease [33,34]. As fibrosis progresses, particularly in older patients with IPF, supportive interventions such as early symptom palliation, pulmonary rehabilitation, and home oxygen therapy become increasingly important for maintaining quality of life [33]. In contrast, pediatric ILD is rare and highly heterogeneous, which likely explains why prior RCTs of iNO have been restricted to adult populations [35]. Treatment of pediatric ILD generally relies on supportive care, along with disease-specific therapy when an underlying etiology is identified [36]. These differences across age groups highlight the importance of identifying clinically and biologically defined subgroups that may be more likely to benefit from iNO rather than applying it broadly to all patients with ILD.

The included studies predominantly enrolled older adults, with mean ages ranging from 64 to 70 years, which may have contributed to the lack of observed improvement of iNO in functional improvements. From a pharmacological perspective, iNO may be most effective in patients with fibrotic ILD who exhibit a pulmonary vascular phenotype characterized by elevated pulmonary vascular resistance with a predominant reversible vasoconstrictive component and sufficiently preserved ventilated lung regions [37]. Aging may attenuate the clinical response to iNO through several mechanisms. First, aging is associated with progressive loss of ventilated alveolar units, limiting the delivery of inhaled NO to target regions [38]. Second, aging accelerates irreversible pulmonary vascular remodeling and fibrotic vascular destruction, thereby reducing the relative contribution of reversible vasoconstriction, which is the primary therapeutic target of iNO [37]. Third, age-related impairment of the nitric oxide–soluble guanylate cyclase–cyclic guanosine monophosphate signaling pathway may further diminish responsiveness to inhaled NO [39]. Collectively, these mechanisms suggest that the predominance of older patients in the included studies may have limited the clinical efficacy of iNO. These findings suggest that future studies should evaluate whether younger patients with a pulmonary vascular phenotype and preserved ventilated lung regions may derive greater benefit from iNO.

This study has several limitations. First, the relatively small pooled sample size may have limited the statistical power to detect differences in the assessed endpoints. Second, three of the four included trials, including the recent phase-3 trial, were conducted by the same research group [6,12,13]. Although the phase-3 trial provided important evidence, its planned sample size was revised downward because of slow recruitment, and differences in findings across trials and wide confidence intervals for several outcomes indicate that residual uncertainty remains [13]. By incorporating all available RCTs, including the independently conducted trial by Freidkin et al., the present meta-analysis provided a more comprehensive synthesis and more precise estimates of treatment effects than any individual trial alone [21]. Third, the certainty of evidence for outcomes of 6MWD and any adverse events was downgraded due to the inclusion of a single randomized trial assessed as having a high risk of bias. Fourth, clinical heterogeneity existed across studies, particularly because Freidkin et al. evaluated a short-duration iNO intervention, whereas the other trials evaluated longer-term treatment [21]. However, because these studies evaluated the same core physiological mechanism of iNO, namely acute selective pulmonary vasodilation rather than long-term pulmonary vascular remodeling, we considered them clinically suitable for synthesis. Finally, this meta-analysis classified PH as confirmed or intermediate-to-high likelihood of PH; therefore, the proportion of patients with confirmed PH may have been lower than estimated. Results may differ in trials restricted to patients with ILD and confirmed PH.

## Conclusion

In this meta-analysis, iNO did not demonstrate benefit in 6MWD or MVPA compared with placebo in patients with advanced and fibrotic ILD. There was no difference in any adverse events between the iNO and placebo groups. Overall, current RCTs suggest no clinically meaningful improvement in functional exercise capacity or daily physical activity with iNO in the study populations included in this meta-analysis. Therefore, iNO cannot currently be recommended as routine therapy for advanced and fibrotic ILD, although its potential role in selected patients with pulmonary vascular phenotypes remains to be clarified.

## Supporting information

S1 File Supplementary Appendix. (DOCX)
