# Supplementary material for: Inhaled nitric oxide in fibrotic and advanced interstitial lung disease: A systematic review and meta-analysis of randomized controlled trials
Source: PLoS One. 2026 Jun 22;21(6):e0351862. doi: 10.1371/journal.pone.0351862 (PMC13286226; doi:10.1371/journal.pone.0351862)

**SUPPLEMENTARY APPENDIX**

**TABLE OF CONTENT**

[**Supplementary Table 1. PRISMA 2020 Main Checklist 2**](#_heading=h.50j4nh1s268i)

[**Supplementary Table 2. Eligibility criteria per included study 5**](#_heading=h.80upss1jok53)

[**Supplementary Table 3. Details of Search Strategy 7**](#_heading=h.alkddzw5z51e)

[**Supplementary Table 4. Endpoint definition per included study 8**](#_heading=h.1jwmvfa1mcw1)

[**Supplementary Table 5. Supplementary Baseline Characteristics 11**](#_heading=)

[**Supplementary Figure 1. Risk of Bias for Parallel RCTs 13**](#_heading=h.d8n4z28nnumi)

[**Supplementary Figure 1A."Traffic light" plot of the domain-level judgments for each study 13**](#_heading=h.be4w3merga9r)

[**Supplementary Figure 1B. Summary of overall weighted bar plot of risk-of-bias judgments within each bias domain. 13**](#_heading=h.7li0wwuc58rj)

[**Supplementary Figure 2. Risk of Bias for Crossover RCTs 14**](#_heading=h.8r5fxshl4qbm)

[**Supplementary Figure 2A."Traffic light" plot of the domain-level judgments for each study 14**](#_heading=h.unksdhpe48sa)

[**Supplementary Figure 2B. Summary of overall weighted bar plot of risk-of-bias judgments within each bias domain. 14**](#_heading=h.z0hhmj90tsat)

[**Supplementary Figure 3. Grading of Recommendations Assessment, Development and Evaluation 15**](#_heading=h.qeks1mvek59h)

[**Supplementary Figure 4. Sensitivity Analysis 16**](#_heading=h.eit20x9wxe3u)

[Supplementary Figure 4A. Leave-one-out for 6MWD endpoint 16](#_heading=h.i8ro8gs4vpwi)

[Supplementary Figure 4B. Leave-one-out for any adverse events endpoint 16](#_heading=h.rjgb73x2vjxq)

[Supplementary Figure 4C. Leave-one-out for MVPA endpoint 16](#_heading=h.1c3n5jrit93n)

#

# **Supplementary Table 1. PRISMA 2020 Main Checklist**

| **Topic** | **No.** | **Item** | **Location where item is reported** |
| --- | --- | --- | --- |
| **TITLE** |  |  |  |
| **Title** | 1 | Identify the report as a systematic review. | Pg .1 |
| **ABSTRACT** |  |  |  |
| **Abstract** | 2 | See the PRISMA 2020 for Abstracts checklist | Pg.2 |
| **INTRODUCTION** |  |  |  |
| **Rationale** | 3 | Describe the rationale for the review in the context of existing knowledge. | Pg.3 |
| **Objectives** | 4 | Provide an explicit statement of the objective(s) or question(s) the review addresses. | Pg.3 |
| **METHODS** |  |  |  |
| **Eligibility criteria** | 5 | Specify the inclusion and exclusion criteria for the review and how studies were grouped for the syntheses. | Pg.4 |
| **Information sources** | 6 | Specify all databases, registers, websites, organizations, reference lists and other sources searched or consulted to identify studies. Specify the date when each source was last searched or consulted. | Pg.4 |
| **Search strategy** | 7 | Present the full search strategies for all databases, registers, and websites, including any filters and limits used. | Supplemental Pg 6 |
| **Selection process** | 8 | Specify the methods used to decide whether a study met the inclusion criteria of the review, including how many reviewers screened each record and each report retrieved, whether they worked independently, and if applicable, details of automation tools used in the process. | Pg.4, Supplemental 4 |
| **Data collection process** | 9 | Specify the methods used to collect data from reports, including how many reviewers collected data from each report, whether they worked independently, any processes for obtaining or confirming data from study investigators, and if applicable, details of automation tools used in the process. | Pg.5 |
| **Data items** | 10a | List and define all outcomes for which data were sought. Specify whether all results that were compatible with each outcome domain in each study were sought (e.g., for all measures, time points, analyses), and if not, the methods used to decide which results to collect. | Pg.4, supplemental 7 |
|  | 10b | List and define all other variables for which data were sought (e.g., participant and intervention characteristics, funding sources). Describe any assumptions made about any missing or unclear information. | Pg.4 |
| **Study risk of bias assessment** | 11 | Specify the methods used to assess risk of bias in the included studies, including details of the tool(s) used, how many reviewers assessed each study and whether they worked independently, and if applicable, details of automation tools used in the process. | Pg.5 |
| **Effect measures** | 12 | Specify for each outcome the effect measure(s) (e.g., risk ratio, mean difference) used in the synthesis or presentation of results. | Pg.5 |
| **Synthesis methods** | 13a | Describe the processes used to decide which studies were eligible for each synthesis (e.g., tabulating the study intervention characteristics and comparing against the planned groups for each synthesis (item 5)). | Pg.5 |
|  | 13b | Describe any methods required to prepare the data for presentation or synthesis, such as handling of missing summary statistics, or data conversions. | Pg.5 |
|  | 13c | Describe any methods used to tabulate or visually display results of individual studies and syntheses. | Pg.5 |
|  | 13d | Describe any methods used to synthesize results and provide a rationale for the choice(s). If meta-analysis was performed, describe the model(s), method(s) to identify the presence and extent of statistical heterogeneity, and software package(s) used. | Pg.5 |
|  | 13e | Describe any methods used to explore possible causes of heterogeneity among study results (e.g., subgroup analysis, meta-regression). | Pg.5 |
|  | 13f | Describe any sensitivity analyses conducted to assess robustness of the synthesized results. | Pg.5 |
| **Reporting bias assessment** | 14 | Describe any methods used to assess risk of bias due to missing results in a synthesis (arising from reporting biases). | Pg.5 |
| **Certainty assessment** | 15 | Describe any methods used to assess certainty (or confidence) in the body of evidence for an outcome. | Pg.5 |
| **RESULTS** |  |  |  |
| **Study selection** | 16a | Describe the results of the search and selection process, from the number of records identified in the search to the number of studies included in the review, ideally using a flow diagram. | Pg.6 |
|  | 16b | Cite studies that might appear to meet the inclusion criteria, but which were excluded, and explain why they were excluded. | Pg.6 |
| **Study characteristics** | 17 | Cite each included study and present its characteristics. | Pg.6 |
| **Risk of bias in studies** | 18 | Present assessments of risk of bias for each included study. | Pg.11 |
| **Results of individual studies** | 19 | For all outcomes, present, for each study: (a) summary statistics for each group (where appropriate) and (b) an effect estimate and its precision (e.g., confidence/credible interval), ideally using structured tables or plots. | Pg.9 |
| **Results of syntheses** | 20a | For each synthesis, briefly summarize the characteristics and risk of bias among contributing studies. | Pg.11 |
|  | 20b | Present results of all statistical syntheses conducted. If meta-analysis was done, present for each the summary estimate and its precision (e.g., confidence/credible interval) and measures of statistical heterogeneity. If comparing groups, describe the direction of the effect. | Pg.9 |
|  | 20c | Present results of all investigations of possible causes of heterogeneity among study results. | Pg.9 |
|  | 20d | Present results of all sensitivity analyses conducted to assess the robustness of the synthesized results. | Pg.9 |
| **Reporting biases** | 21 | Present assessments of risk of bias due to missing results (arising from reporting biases) for each synthesis assessed. | Pg.9 |
| **Certainty of evidence** | 22 | Present assessments of certainty (or confidence) in the body of evidence for each outcome assessed. | Pg.11 |
| **DISCUSSION** |  |  |  |
| **Discussion** | 23a | Provide a general interpretation of the results in the context of other evidence. | Pg.11 |
|  | 23b | Discuss any limitations of the evidence included in the review. | Pg.11,12,13 |
|  | 23c | Discuss any limitations of the review processes used. | Pg.11,12,13 |
|  | 23d | Discuss implications of the results for practice, policy, and future research. | Pg.11,12,13 |
| **OTHER INFORMATION** |  |  |  |
| **Registration and protocol** | 24a | Provide registration information for the review, including register name and registration number, or state that the review was not registered. | Pg.4 |
|  | 24b | Indicate where the review protocol can be accessed, or state that a protocol was not prepared. | Pg.4 |
|  | 24c | Describe and explain any amendments to information provided at registration or in the protocol. | Pg.4 |
| **Support** | 25 | Describe sources of financial or non-financial support for the review, and the role of the funders or sponsors in the review. | Pg.16 |
| **Competing interests** | 26 | Declare any competing interests of review authors. | Pg.16 |
| **Availability of data, code and other materials** | 27 | Report which of the following are publicly available and where they can be found template data collection forms; data extracted from included studies; data used for all analyses; analytic code; any other materials used in the review. | Pg.16 |

# **Supplementary Table 2. Eligibility criteria per included study**

| **Study and Year** | **Inclusion Criteria** | **Exclusion Criteria** |
| --- | --- | --- |
| Freidkin, et al 2024 | ・Patients with COPD, GOLD stage 3–4  ・Patients with IPF with: FVC < 80% predicted, and DLCO < 60% predicted  ・Ambulatory patients able to perform a 6MWT | ・Moderate to severe systolic heart failure, defined as LVEF ≤40%  ・Severe peripheral vascular disease or scleroderma  ・Inability to perform a 6-minute walk test |
| King, et al 2022 | ・Radiologic criteria 　・HRCT within 6 months before screening 　・Findings consistent with interstitial lung 　　disease 　・Diagnosis confirmed according to 　　　　ATS/ERS/JRS/ALAT guidelines  ・Eligible ILD diagnoses 　・Idiopathic pulmonary fibrosis　 　・Idiopathic nonspecific interstitial 　　　　　pneumonia 　 　・Respiratory bronchiolitis–ILD 　・Desquamative interstitial pneumonia 　　・Cryptogenic organizing pneumonia 　 　・Acute interstitial pneumonia 　・Idiopathic lymphoid interstitial 　　　　　　pneumonia 　・Idiopathic pleuroparenchymal 　　　　　　fibroelastosis  　・Unclassifiable idiopathic interstitial 　　　　pneumonias 　・Chronic hypersensitivity pneumonitis 　・Occupational lung disease 　・Connective tissue disease–associated ILD  　・Interstitial pneumonia with autoimmune 　　features  ・Use of supplemental oxygen via nasal 　　　cannula for ≥4 weeks, including use limited 　to exertion  ・6MWD between 100 and 400 meters at 　　screening and baseline/randomization visits  ・World Health Organization Functional Class II–IV  ・Forced vital capacity ≥40% predicted within 　60 days prior to screening  ・Age 18–80 years at screening | ・Women of childbearing potential who are: 　・Pregnant or breastfeeding at screening 　・Planning to become pregnant 　・Unwilling to use appropriate 　　　　　　　contraception if sexually active during 　　　the study and for at least 30 days after 　　　discontinuation of the study drug  ・Heart failure 　・Heart failure with reduced ejection 　　　　fraction with LVEF <40%, or 　・Severe heart failure with preserved 　　　　ejection fraction  ・History of sarcoidosis  ・History of chronic thromboembolic 　　　　pulmonary hypertension (CTEPH; WHO 　　Group 4 PH)  ・Smoking within 3 months prior to screening 　or unwillingness to abstain from smoking 　throughout the study  ・Body mass index >40 kg/m² at 　　　screening  ・Intermittent uncontrolled atrial fibrillation, 　as judged by the Principal Investigator  ・Severe hepatic impairment, as judged by the 　Principal Investigator  ・Severe renal impairment, defined as 　　　　estimated creatinine clearance <30 mL/min 　calculated using the CKD-EPI 2009 　　　　equation, at screening |
| Nathan, et al 2020 | Same criteria as King, et al 2022 | Same criteria as King, et al 2022 |
| Nathan, et al 2024 | Same criteria as King, et al 2022 | Same criteria as King, et al 2022 |

6MWD, six-minute walk distance; 6MWT, six-minute walk test; ATS, American Thoracic Society; CKD-EPI, Chronic Kidney Disease Epidemiology Collaboration; COPD, chronic obstructive pulmonary disease; CTEPH, chronic thromboembolic pulmonary hypertension; DLCO, diffusing capacity of the lung for carbon monoxide; ERS, European Respiratory Society; FVC, forced vital capacity; GOLD, Global Initiative for Chronic Obstructive Lung Disease; HRCT, high-resolution computed tomography; ILD, interstitial lung disease; IPF, idiopathic pulmonary fibrosis; JRS, Japanese Respiratory Society; LVEF, left ventricular ejection fraction; PH, pulmonary hypertension

# **Supplementary Table 3. Details of Search Strategy**

| **Search Strategy for each database** | |
| --- | --- |
| **MEDLINE**  **(Pubmed)** | ("Lung Diseases, Interstitial" OR "pulmonary fibrosis" OR "pulmonary fibrosis"[Mesh] ) AND ("Nitric Oxide" OR "Nitric Oxide"[Mesh]) AND (randomized controlled trial[pt] OR controlled clinical trial[pt] OR clinical trials as topic[mesh:noexp] OR trial[ti] OR random*[tiab] OR placebo*[tiab]) |
| **EMBASE** | ('lung diseases, interstitial' OR 'pulmonary fibrosis' OR 'lung fibrosis'/exp) AND ('nitric oxide' OR 'nitric oxide'/exp) AND ('randomized controlled trial':it OR 'controlled clinical trial':it OR 'clinical trial (topic)'/de OR 'trial':ti OR 'random*':ti,ab,kw OR 'placebo*':ti,ab,kw) |
| **CENTRAL**  **(Cochrane)** | ("Lung Diseases, Interstitial" OR "pulmonary fibrosis") AND ("Nitric Oxide") |
| **ClinicalTrials.Gov** | ("Lung Diseases, Interstitial" OR "pulmonary fibrosis") AND ("Nitric Oxide") |

# **Supplementary Table 4. Endpoint definition per included study**

| **Study and Year** | **6MWD** | **Any adverse events** | **MVPA** |
| --- | --- | --- | --- |
| Freidkin, et al 2024 | For placebo 6MWTs, the iNO generator was charged with a placebo cartridge. For iNO 6MWTs, the generator was charged with nitric oxide cartridges. There was no visible difference between placebo and nitric oxide cartridges; both gases were odorless and colorless.  Administration of iNO or placebo began 30 minutes before the start of the 6MWT. Treatment continued until completion of the test.  All 6MWTs were performed under investigator supervision. Tests were conducted in accordance with European Respiratory Society/American Thoracic Society guidelines.  Heart rate and peripheral oxygen saturation were recorded continuously from baseline through 5 minutes after completion of the 6MWT. | NR | NR |
| King, et al 2022 | 6MWD was defined as the total distance walked in meters during a standardized 6MWT.  6MWT were captured at baseline and at  4-week intervals throughout the study  period.  6MWTs were conducted and adapted from the European Respiratory  Society/American Thoracic Society  guidelines.  The INOpulse or placebo device was attached to the oxygen cylinder for all 6MWTs conducted during the study.  Patients were required to pull the same-sized oxygen tank and cart at the same oxygen  flow rate for all their 6MWTs conducted  throughout the study.  Peripheral oxygen saturation (SpO2) was continuously monitored via pulse oximetry during all 6MWTs. | Adverse event was defined as any untoward medical occurrence in a participant who received study treatment, regardless of causal relationship to the investigational product.  Adverse events were collected at each study visit and continuously throughout the safety follow-up period. | MVPA was defined as the total number of minutes per day spent in moderate or vigorous activity, identified using established ActiGraph cut-points.  Minute-level activity counts ≥1952 counts/min were classified as moderate activity and counts ≥5725 counts/min as vigorous activity.  Daily MVPA was calculated as the sum of minutes spent in moderate and vigorous activity.  Only compliant days, defined as ≥600 minutes of awake wear time, were included.  Monthly MVPA values were calculated by averaging daily MVPA across all compliant days within the month, with a compliant month defined as ≥14 compliant days. |
| Nathan, et al 2020 | Serial 6MWTs were performed according to study-specific and American Thoracic Society guidelines at screening, randomization/baseline, and weeks 4 and 8 of the blinded treatment period.  Subjects were required to pull the same oxygen tank and cart using the same oxygen flow rate, and peripheral oxygen saturation (SpO2) was monitored continuously via pulse oximeter (Nonin WristOx2 3150/4000, Tiger Medical, Inc.; Masimo SET Rainbow).  The INOpulse device (Bellerophon Therapeutics) was attached to the oxygen cylinder for all 6MWT conducted throughout the study. The SpO2 nadir and change from baseline were recorded. | Adverse events are defined as any untoward medical occurrences in patients or clinical investigation subjects receiving a pharmaceutical product, which may include unfavorable and unintended signs, symptoms, or diseases that are temporally associated with the use of the product, regardless of a causal relationship. | MVPA was defined as the total number of minutes per day spent in moderate or vigorous activity, as assessed by a medical-grade tri-axial actigraphy monitor.  Minute-level activity counts ≥1952 counts/min were classified as moderate activity and counts ≥5725 counts/min as vigorous activity.  Daily MVPA was calculated as the sum of minutes spent in moderate and vigorous activity. |
| Nathan, et al 2024 | 6MWD was defined as the total distance walked in meters during a standardized 6MWT.  6MWTs were conducted and adapted from the European Respiratory  Society/American Thoracic Society  guidelines.  The INOpulse or placebo device was attached to the oxygen cylinder for all 6MWTs conducted during the study.  Patients were required to pull the same-sized oxygen tank and cart at the same oxygen  flow rate for all their 6MWTs conducted  throughout the study.  Peripheral oxygen saturation (SpO2) was continuously monitored via pulse oximetry during all 6MWTs. | Adverse events are defined as any untoward medical occurrences in patients or clinical investigation subjects receiving a pharmaceutical product, which may include unfavorable and unintended signs, symptoms, or diseases that are temporally associated with the use of the product, regardless of a causal relationship. | MVPA was defined as the total number of minutes per day spent in moderate or vigorous activity, as assessed by a medical-grade tri-axial actigraphy monitor.  Minute-level activity counts ≥1952 counts/min were classified as moderate activity and counts ≥5725 counts/min as vigorous activity.  Daily MVPA was calculated as the sum of minutes spent in moderate and vigorous activity.  Only compliant bi-weeks, defined as ≥8 compliant days including at least 2 weekend days, were included in the primary analysis. |

6MWD, six-minute walk distance; 6MWT, six-minute walk test; AE, adverse event; ATS, American Thoracic Society; ERS, European Respiratory Society; iNO, inhaled nitric oxide; MVPA, moderate-to-vigorous physical activity; NO, nitric oxide; NR, not reported; SpO₂, peripheral oxygen saturation.

# **Supplementary Table 5. Supplementary Baseline Characteristics**

| **Study and**  **year** | **Study Type** | **Country** | **Recruitment**  **period** | **White** | **BMI,**  **kg/m2** | **MVPA,**  **min/d** | **CTD-ILD** | **iNSIP** |
| --- | --- | --- | --- | --- | --- | --- | --- | --- |
| **Freidkin**  **2024** | Crossover  RCT | Israel | November 2019-  January 2023 | NA | 28.0 | NA | 2  (4.5) | 5  (11.4) |
| **King**  **2022** | RCT | United States | January 2019-  July 2019 | 40  (90.9) | 31.7 | 75.0 | NA | 6  (13.6) |
| **Nathan**  **2024** | RCT | United States | November 2020-  December 2022 | 117  (80.7) | 29.2 | 67.1 | 23  (15.9) | 13  (9.0) |
| **Nathan**  **2020** | RCT | United States | Not documented | NA | NA | NA | NA | 2  (4.9) |

BMI, body mass index; CTD-ILD, connective tissue disease–associated interstitial lung disease; iNSIP, idiopathic nonspecific interstitial pneumonia; MVPA, moderate-to-vigorous physical activity; NA, not available; RCT, randomized controlled trial

Categorical data were reported as counts and frequencies (%).

Continuous data reported as mean or median

# **Supplementary Fig. 1 Risk of Bias for Parallel RCTs**

# **Supplementary Fig. 1A"Traffic light" plot of the domain-level judgments for each study**

# **
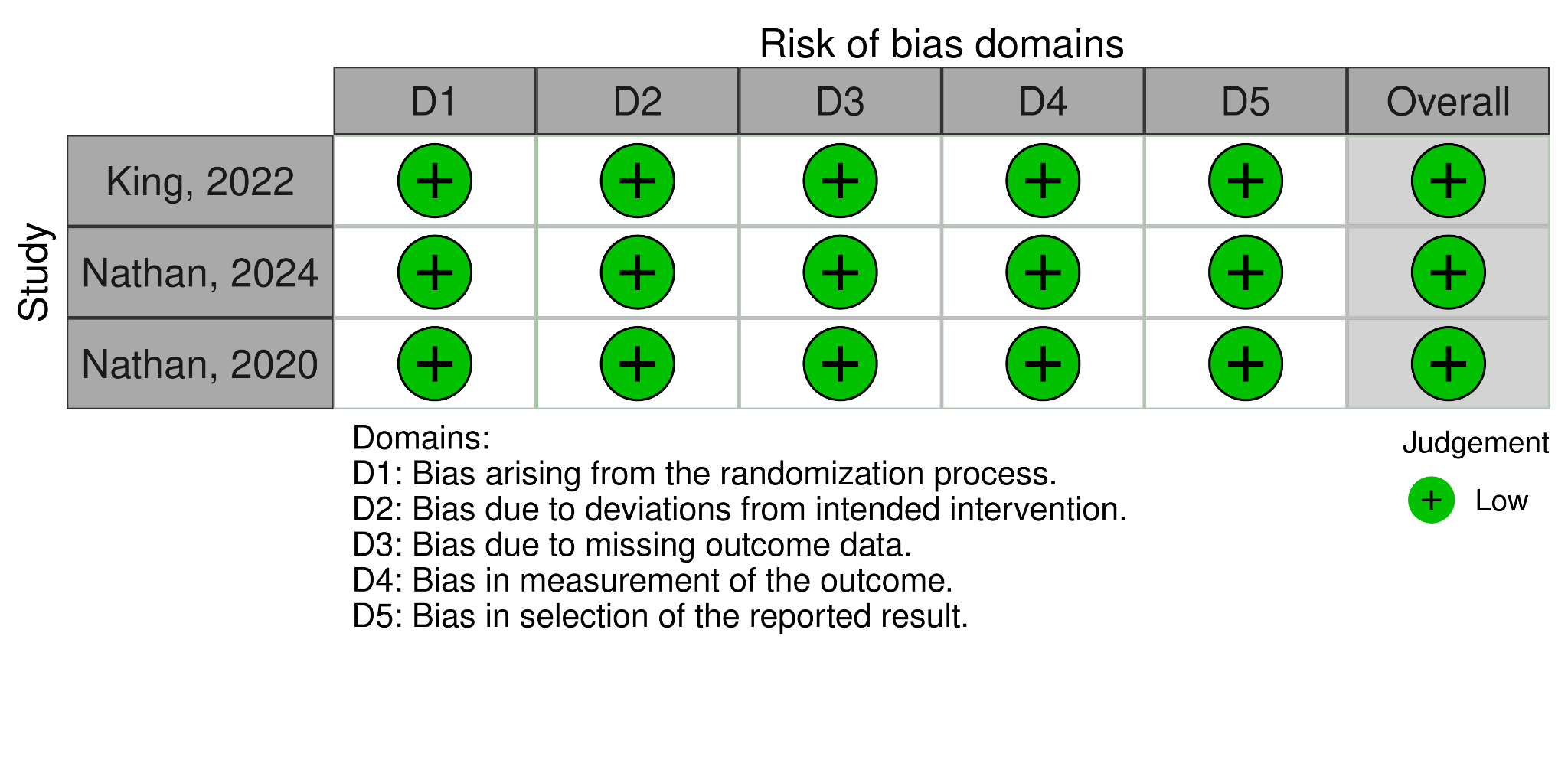
**

# **Supplementary Fig. 1B Summary of overall weighted bar plot of risk-of-bias judgments within each bias domain.**

# **
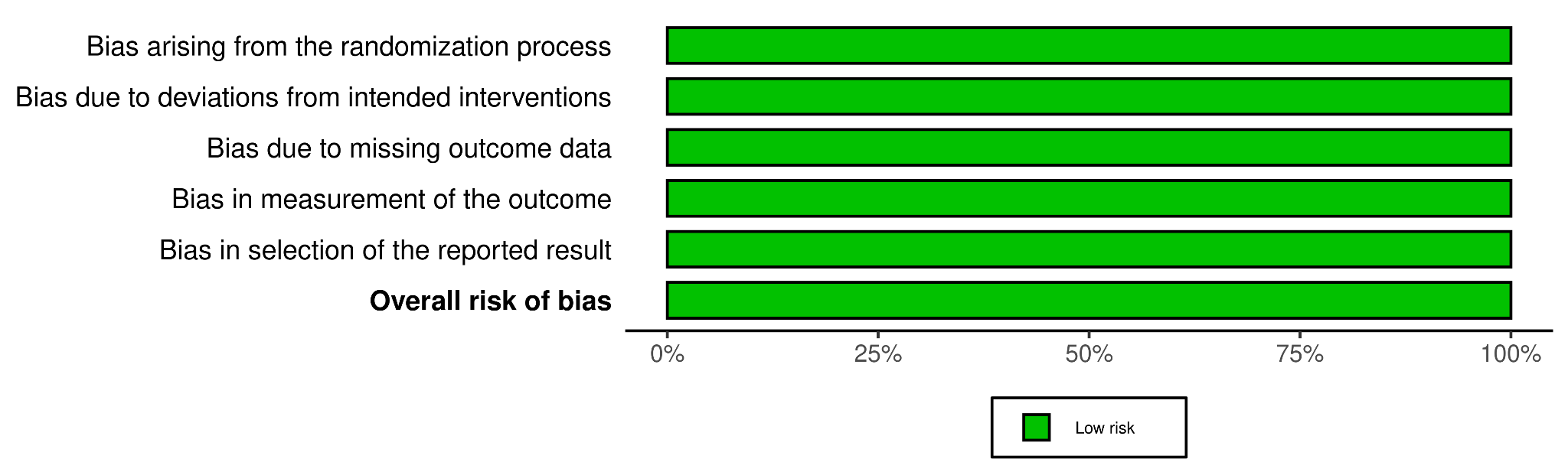
**

# **Supplementary Fig. 2 Risk of Bias for Crossover RCTs**

# **Supplementary Fig. 2A "Traffic light" plot of the domain-level judgments for each study**

# **
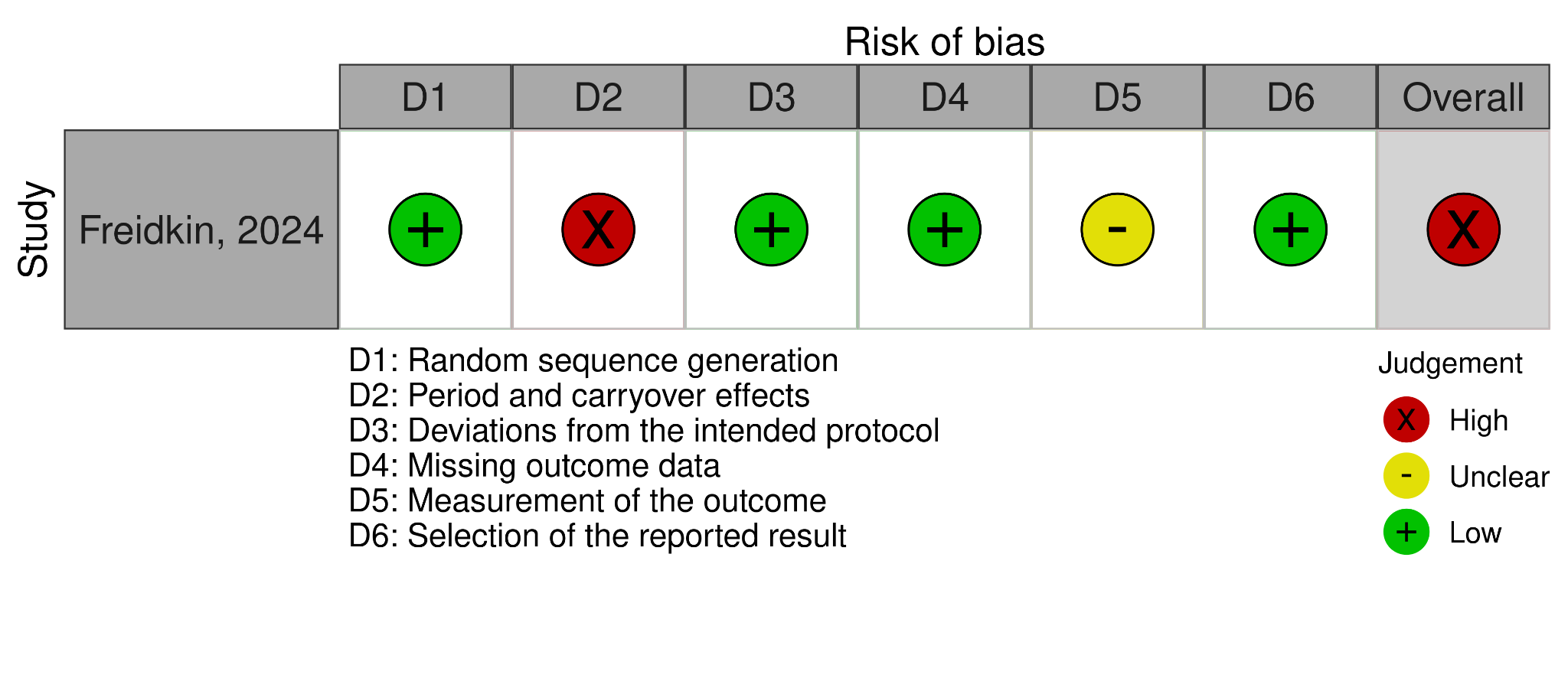
Supplementary Fig. 2B Summary of overall weighted bar plot of risk-of-bias judgments within each bias domain.**

# **
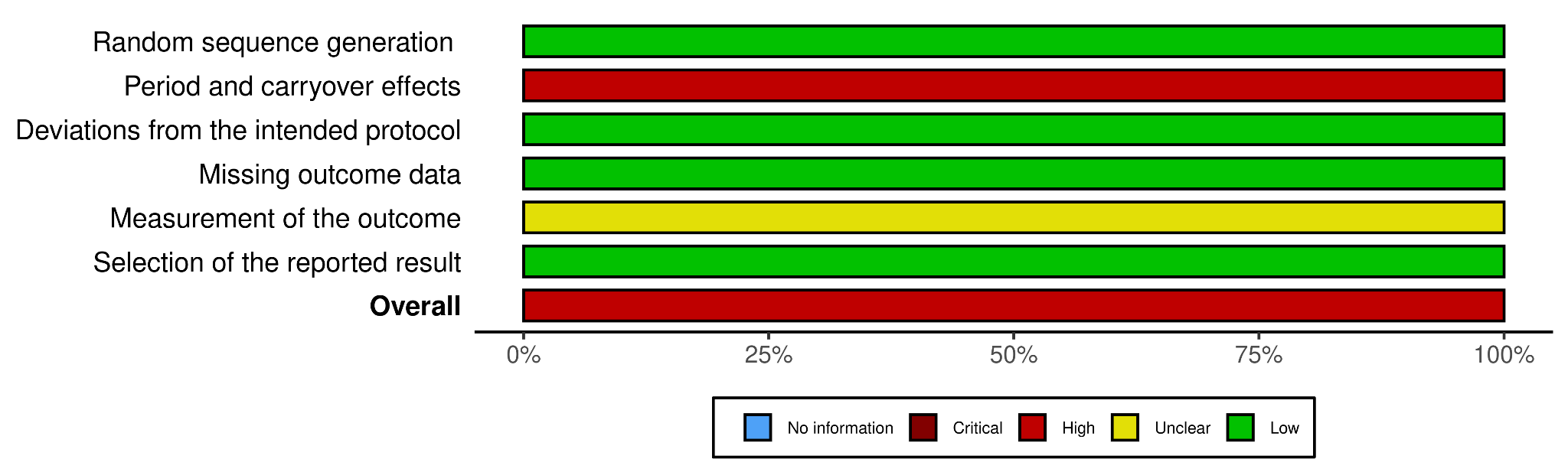
**

# **Supplementary Fig. 3 Grading of Recommendations Assessment, Development and Evaluation**

​
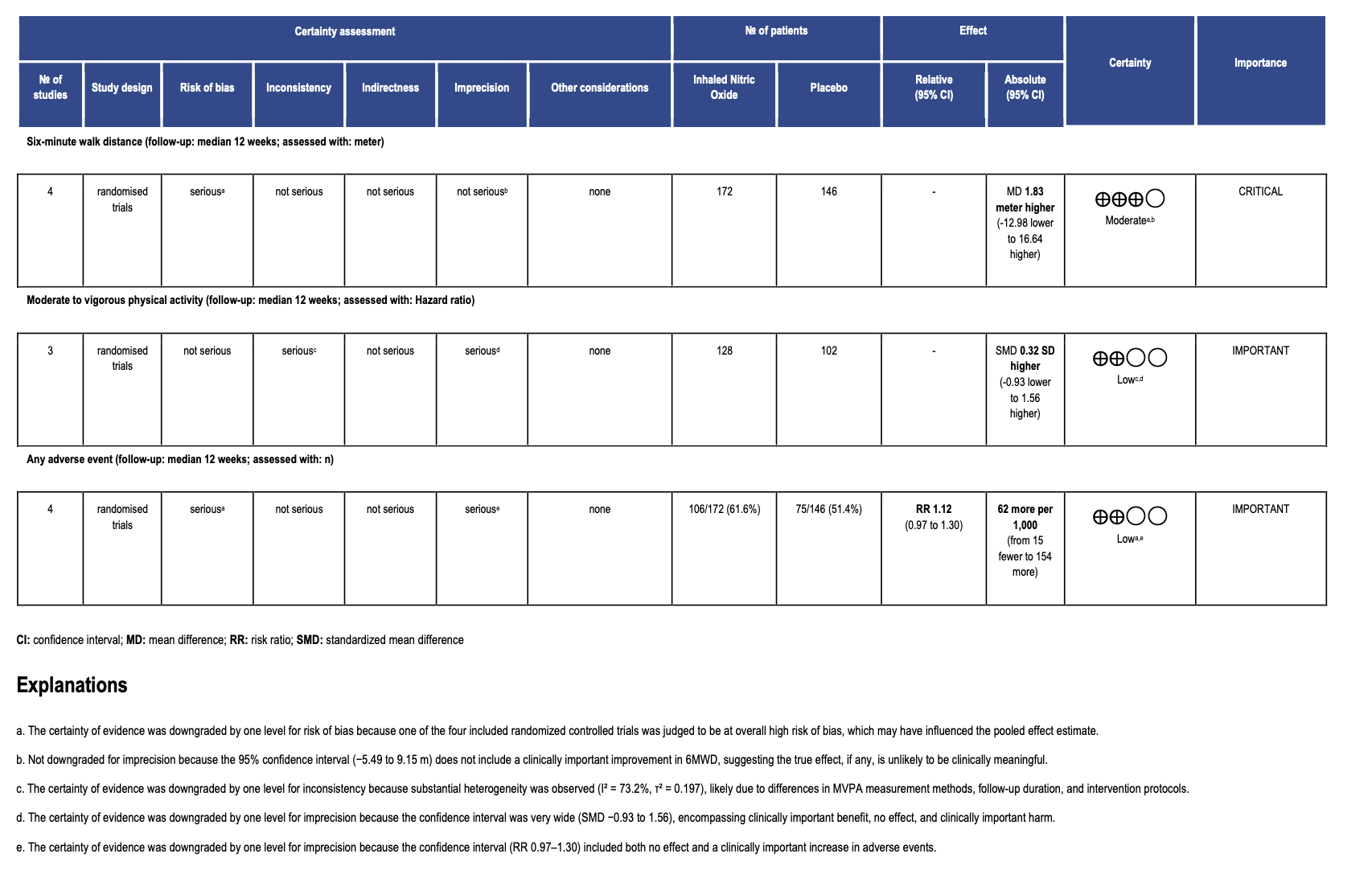


# **Supplementary Fig. 4 Sensitivity Analysis**

## Supplementary Fig. 4A Leave-one-out for 6MWD endpoint


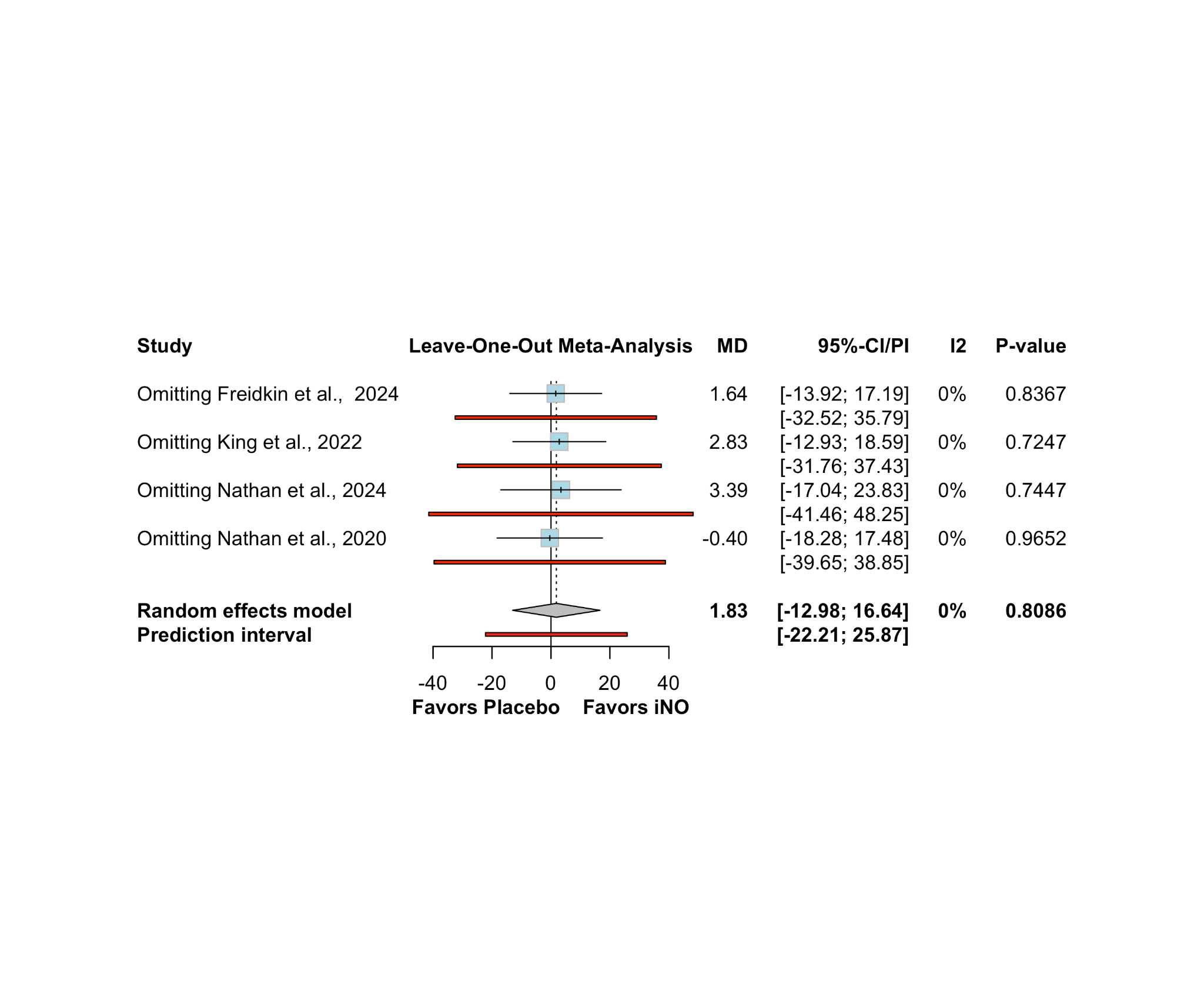


## Supplementary Fig. 4B. Leave-one-out for any adverse events endpoint


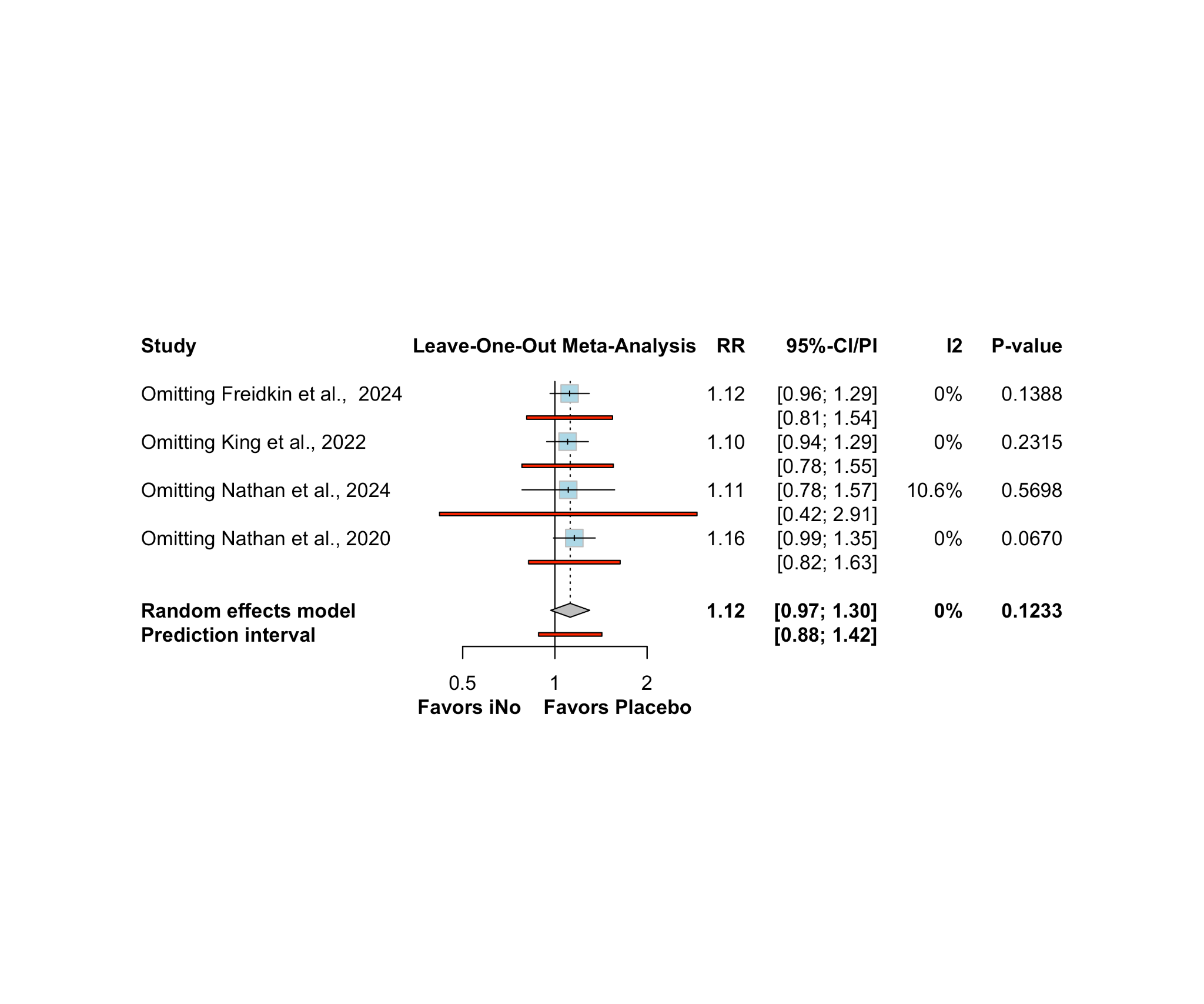


## Supplementary Fig. 4C. Leave-one-out for MVPA endpoint


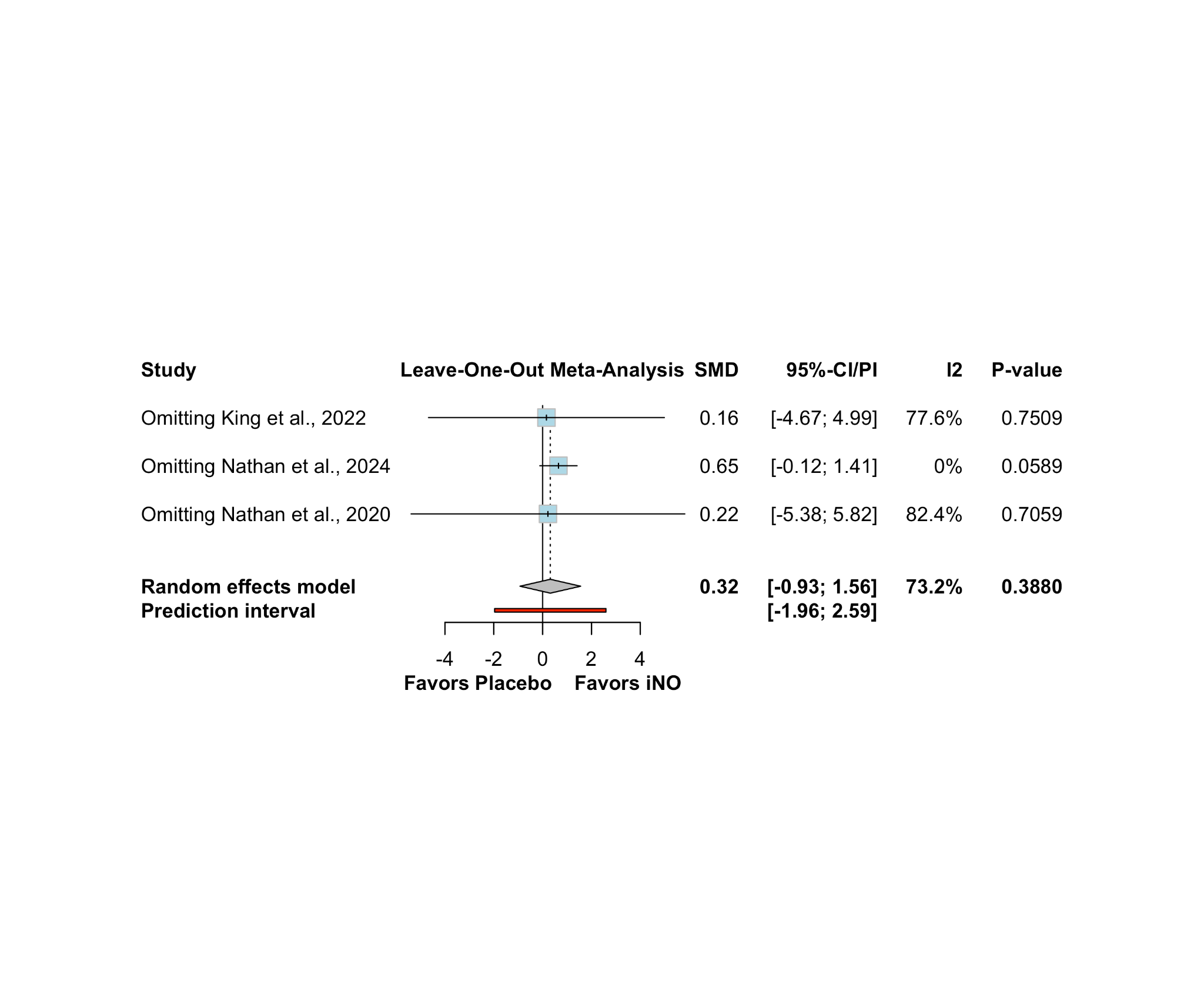

Supplement: S1 File — (DOCX) [file pone.0351862.s001.docx]
