# Supplementary material for: Inhaled nitric oxide in fibrotic and advanced interstitial lung disease: A systematic review and meta-analysis of randomized controlled trials
Source: PLoS One. 2026 Jun 22;21(6):e0351862. doi: 10.1371/journal.pone.0351862 (PMC13286226; doi:10.1371/journal.pone.0351862)
Supplement: Attachment — Submitted filename: Response to Reviewers.docx [file pone.0351862.s003.docx]

**Response to Reviewers**

PONE-D-26-11568

Inhaled Nitric Oxide in Fibrotic and Advanced Interstitial Lung Disease: A Systematic Review and Meta-Analysis of Randomized Controlled Trials

Page and line numbers cited in this response refer to the unmarked revised manuscript labeled “Manuscript”

**Reviewer #1:
Comment 1.** Inclusion of current treatment modalities across different age groups of ILD

**Reply:** Thank you for this helpful comment. We have added a discussion of current treatment modalities for ILD across different age groups and interpreted these considerations in relation to the findings of our meta-analysis. We believe that providing this clinical context helps readers better understand the current therapeutic landscape of ILD and the potential role of iNO as an adjunctive therapy in selected populations.

**Change in the text:** We added a summary of current treatment modalities across different age groups of ILD and discussed how this context relates to the interpretation of our findings. **(Page 15, Line 255-266)**

**Comment 2.** The authors could justify how the analysis was performed to include both short (20 min)and long term (weeks) of iNO exposure. The authors could include the effects of the same as well as the advantages and disadvantages of short and long term iNO exposure from a clinician perspective in the discussion.

**Reply:** Thank you for this important comment. We agree that the study by Freidkin et al. differed from the trials by Nathan’s group in terms of treatment duration. However, the primary mechanism of iNO is not considered to be long-term improvement in pulmonary vascular remodeling, but rather acute selective pulmonary vasodilation that increases blood flow to ventilated lung regions during inhalation. Therefore, although the exposure duration differed across studies, we considered them to be evaluating the same core physiological mechanism and therefore suitable for quantitative synthesis. In addition, leave-one-out sensitivity analyses excluding Freidkin et al. showed similar results without meaningful changes in heterogeneity, supporting the appropriateness of including this study in the pooled analysis.

From a clinical perspective, compared with short-term administration, prolonged iNO therapy may offer the potential for more sustained pulmonary hemodynamic effects, including reductions in pulmonary artery pressure and pulmonary vascular resistance. However, prolonged therapy also involves additional clinical considerations, including potential worsening oxygenation or hemodynamic instability with abrupt discontinuation, the need for careful weaning, device-related burden, adherence challenges, and cost.

**Change in the text:** We added a limitation and discussion explaining the rationale for including both short-duration and longer-term iNO studies in the quantitative synthesis.  **(Page 14, Line 229-237)** **(Page 16, Line 295-300)**

**Comment 3.** The authors have stated “For MVPA, the certainty of evidence was downgraded for serious inconsistency due to substantial heterogeneity”. Please discuss the possible reasons for the same in previous RCTs.

**Reply:** Notably, in the leave-one-out analysis, exclusion of Nathan et al. (2024) resulted in complete resolution of heterogeneity (I² = 0%), suggesting that this study was the primary contributor to the observed variability. One potential explanation for the observed heterogeneity is the difference in baseline prevalence of pulmonary hypertension across studies, with a lower proportion in Nathan et al. (2024).

**Change in the text:** We added a discussion of the potential reasons for heterogeneity in MVPA, including the results of the leave-one-out analysis and differences in baseline pulmonary hypertension prevalence across the included RCTs. **(Page 13, Line 224-226)**

**Comment 4.** The majority of the population were adults over 60 years and male. Please discuss the same in the context of ILD and iNO therapy. Does the conclusion of no clinical improvement be attributed to the population considered. Is iNO administered to any other age group?

**Reply:**  Thank you for this important comment. We agree that the demographic characteristics of the included populations are important when interpreting our findings. The included RCTs predominantly enrolled older adults, with mean ages ranging from 64 to 70 years, and most participants were male. Therefore, our conclusion of no clinically meaningful improvement should be interpreted in the context of the populations enrolled in the available RCTs, rather than generalized to all patients with ILD. We added a discussion explaining that older age may attenuate the response to iNO through several mechanisms, including loss of ventilated alveolar units, irreversible pulmonary vascular remodeling, fibrotic vascular destruction, and age-related impairment of the nitric oxide–soluble guanylate cyclase–cyclic guanosine monophosphate signaling pathway. These mechanisms may reduce the likelihood that acute selective pulmonary vasodilation translates into functional improvement.

**Change in the text:** We added a discussion of the age and sex distribution of the included populations and explained how older age may have limited the clinical response to iNO. We also clarified that the conclusion should be interpreted in the context of the populations enrolled in the current RCTs and added discussion regarding the lack of pediatric ILD data.  **(Page 15,16, Line 267-283)**

**Comment 5.** Would the author recommend the feasibility of inclusion of iNO in routine therapy – in light of advantages and disadvantages considering no safety issue. What are the drawbacks for iNO considering routine therapy wherever considered. Why were the same restricted to adults in RCTs.

**Response:** Based on the current evidence, we would not recommend iNO as routine therapy for all patients with fibrotic ILD. However, it may be considered in carefully selected patients, particularly those who are younger than the populations enrolled in prior RCTs, have elevated PVR with a substantial reversible vasoconstrictive component, and retain sufficiently ventilated lung regions. The main barriers to routine use include uncertain clinical benefit, cost, device burden, and the need for careful phenotyping. Prior RCTs were likely restricted to adults because fibrotic ILD predominantly affects older adults and pediatric ILD is rare and clinically heterogeneous.

**Change in the text:** We added a discussion of the age distribution of the included populations and explained how older age may have limited the clinical response to iNO. **(Page 15,16, Line 272-283)** We also clarified that the conclusion should be interpreted in the context of the populations enrolled in the current RCTs **(Page 17, Line 308-313)** and added discussion regarding the lack of pediatric ILD data.**(Page 15, Line 260-264)**

**Comment 6.** To benefit the reader, the final conclusion could include authors perspective and recommendation on use of iNO for ILD

**Response:** Thank you for this helpful suggestion. We revised the Conclusion to include our clinical perspective on the use of iNO in ILD. Based on the current evidence, iNO cannot be recommended as routine therapy for patients with advanced and fibrotic ILD. However, its potential role in carefully selected patients, particularly those with pulmonary vascular phenotypes, remains to be clarified.

**Change in the text:** We revised the Conclusion to clarify that iNO cannot currently be recommended as routine therapy for advanced and fibrotic ILD, while noting that its potential role in selected patients with pulmonary vascular phenotypes remains an important area for future investigation. **(Page 17, Line 310-313)**

**Reviewer #2:**
MAJOR COMMENTS:
**Comment 1. Rationale of the study**
The authors state that previous RCTs have demonstrated increases in MVPA, alongside improvements in dyspnea, quality-of-life measures, and oxygenation. However, a phase-3 trial in patients with fILD found no significant differences in activity level, 6MWD or patient-reported outcomes. They justify their study on the basis that the available evidence regarding iNO in ILD is inconsistent and limited to small-scale trials.
King et al. conducted a phase 2 exploratory randomized controlled trial and found that INOpulse was well tolerated and associated with the maintenance of physical activity and an improvement in symptoms in patients with ILD requiring supplemental oxygen (AnnalsATS, 2021). They then conducted a randomized, double-blind, placebo-controlled trial to validate the benefit of ambulatory iNO in patients with fILD requiring supplemental oxygen, concluding that there was no demonstrable benefit to iNO in patients with the disorder (AnnalsATS, 2024).
Are a further systematic review and meta-analysis necessary?

**Response:** Thank you for this important comment. We acknowledge that a phase 3 trial (Nathan et al., 2024) has provided important evidence and may appear to address the question of efficacy. However, the trial’s planned sample size was revised downward from 300 to 140 participants because of slow recruitment. Although the revised sample size was justified based on updated assumptions from the phase 2 data, the final evidence base remains relatively limited when considered across all randomized trials. Although the phase 3 trial reported neutral findings, earlier trials suggested potential benefits in MVPA. Rather than focusing solely on statistical significance, integrating these data allows for a more precise estimation of the effect size and its uncertainty. Similarly, outcomes such as 6MWD and any adverse events consistently showed no statistically significant differences across individual trials. However, the confidence intervals in these studies remain wide. By pooling all available randomized data, this meta-analysis reduces uncertainty, improves the precision of effect estimates. Given the limited number of trials and total sample size, such quantitative synthesis remains essential to strengthen the overall certainty of evidence. Therefore, we believe this study provides meaningful additional value beyond individual trials.

**Changes in the text:** We clarified the rationale for conducting this systematic review and meta-analysis in the Introduction and added discussion in the Limitations section explaining that, despite the important contribution of the recent phase III trial, the overall randomized evidence base remains limited and residual uncertainty remains. **(Page 5, Line 63-66 and Page 16, Line 285-293)**

**Comment 2. Studies included in review**
The authors included four RCTs in this systematic review and meta-analysis. The first RCT was conducted by Nathan et al. and enrolled 41 patients. The second RCT was reported by King et al., although Nathan contributed to the study design, data analysis, and manuscript preparation and revision. The third RCT was performed by Nathan et al. to validate the benefits of ambulatory iNO. Thus, these three RCTs were all conducted by the same group. The fourth study was conducted independently of the others. However, this study only included 44 patients. Is it therefore reasonable to conduct a systematic review and meta-analysis?

**Response:** Thank you for this great comment. We acknowledge that three of the four included trials, including the recent phase III trial, were conducted by the same research group. As noted in our response to Comment 1, although the phase III trial provided important evidence, the overall randomized evidence base remains limited because the trial’s planned sample size was revised downward and the total number of available RCTs remains small. We also agree that the independently conducted study by Freidkin et al. had a small sample size of 44 patients. However, given that only four RCTs are currently available and the total sample size of the three trials from Nathan’s group was also modest, we believe that including Freidkin et al. provided valuable additional information and allowed a more comprehensive synthesis of all randomized evidence. Therefore, we considered it reasonable and clinically informative to conduct a systematic review and meta-analysis, while explicitly acknowledging the limited number of trials and the concentration of studies from a single research group as important limitations.

**Changes in the text:** We added text to the Limitations section acknowledging that three of the four included trials, including the recent phase III trial, were conducted by the same research group. We also clarified that, despite this limitation, incorporating all available RCTs, including the independently conducted study by Freidkin et al., allowed a more comprehensive synthesis of the limited randomized evidence. **(Page 14,15, Line 248-254)**

**Comment 3. Baseline patient and study characteristics**
According to Table 1, Freidkin et al. administered iNO for 20 minutes once. In contrast, Nathan’s group delivered NO consistently for 8 to 16 weeks (at a daily usage rate of >12 hours). Are the results of the study by Freidkin et al. comparable to those of Nathan’s group?

**Response:** We agree that the study by Freidkin et al. differed from the trials by Nathan’s group in terms of treatment duration. However, because these studies shared the same core physiological mechanism of iNO—acute selective pulmonary vasodilation in ventilated lung regions rather than long-term pulmonary vascular remodeling—we considered them clinically suitable for synthesis, while acknowledging clinical heterogeneity in treatment duration. Therefore, we performed leave-one-out sensitivity analyses, which showed that the overall conclusions were robust to exclusion of Freidkin et al. This suggests that including this clinically distinct trial did not materially alter the pooled findings.

**Change in the text:** We added a discussion explaining the rationale for including both short-duration and longer-term iNO studies in the quantitative synthesis.  **(Page 16, Line 295-300)**

MINOR COMMENTS:

**Comment 1.** Supplementary Table 5

The study by Freidkin et al. was conducted in Israel rather than the United States.

**Response:**
Thank you for pointing this out.

**Changes in the text:**
We corrected the country of the Freidkin et al. study in **Supplementary Table 5**.

**Journal Requirements:**

1. When submitting your revision, we need you to address these additional requirements.

Please ensure that your manuscript meets PLOS ONE's style requirements, including those for file naming. The PLOS ONE style templates can be found at

https://journals.plos.org/plosone/s/file?id=wjVg/PLOSOne_formatting_sample_main_body.pdf and

https://journals.plos.org/plosone/s/file?id=ba62/PLOSOne_formatting_sample_title_authors_affiliations.pdf

**Response:** Thank you for this instruction. We have reviewed the PLOS ONE style requirements and revised the manuscript formatting and file names accordingly.

2. Your ethics statement should only appear in the Methods section of your manuscript. If your ethics statement is written in any section besides the Methods, please move it to the Methods section and delete it from any other section. Please ensure that your ethics statement is included in your manuscript, as the ethics statement entered into the online submission form will not be published alongside your manuscript.

**Response:** Thank you for this instruction. We moved the ethics statement to the Materials and methods section and removed it from other sections. **(Page 8, Line 142-145)**

3. Please include captions for your Supporting Information files at the end of your manuscript, and update any in-text citations to match accordingly. Please see our Supporting Information guidelines for more information: http://journals.plos.org/plosone/s/supporting-information.

**Response:** We added captions for all Supporting Information files at the end of the manuscript and reviewed the in-text citations to ensure that they match the corresponding Supporting Information files. **(Page 22, Line 454-463)**

4. Please upload a new copy of Figure S3, as the detail is not clear. Please follow the link for more information:  https://journals.plos.org/plosone/s/figures

**Response:** We uploaded a new copy of Figure S3 with improved image quality to enhance readability.

5. If the reviewer comments include a recommendation to cite specific previously published works, please review and evaluate these publications to determine whether they are relevant and should be cited. There is no requirement to cite these works unless the editor has indicated otherwise.

**Response:** No changes were made in response to this item because no specific additional references were recommended by the reviewers.
