# Supplementary material for: Inhaled nitric oxide in fibrotic and advanced interstitial lung disease: A systematic review and meta-analysis of randomized controlled trials
Source: PLoS One. 2026 Jun 22;21(6):e0351862. doi: 10.1371/journal.pone.0351862 (PMC13286226; doi:10.1371/journal.pone.0351862)
Supplement: Peer review history

# Peer review: Decision Letter 0

**DOI:** 10.1371/journal.pone.0351862.r001

**Authors:** Selvakumar Subbian

30 Apr 2026

PONE-D-26-11568 Inhaled Nitric Oxide in Fibrotic and Advanced Interstitial Lung Disease: A Systematic Review and Meta-Analysis of Randomized Controlled Trials PLOS One

Dear Dr. Nakahara,

Thank you for submitting your manuscript to PLOS ONE. After careful consideration, we feel that it has merit but does not fully meet PLOS ONE’s publication criteria as it currently stands. Therefore, we invite you to submit a revised version of the manuscript that addresses the points raised during the review process.

==============================

**One of the reviewers questioned the need for this systematic review and the value of the study. I suggest the authors to highlight the significance of this study and emphasize the new findings that have not been reported previously. Add additional insights into the discussion and make compelling arguments to justify this study.**

==============================

Please submit your revised manuscript by Jun 14 2026 11:59PM. If you will need more time than this to complete your revisions, please reply to this message or contact the journal office at plosone@plos.org. When you're ready to submit your revision, log on to https://www.editorialmanager.com/pone/ and select the 'Submissions Needing Revision' folder to locate your manuscript file.

Please include the following items when submitting your revised manuscript:

- A letter that responds to each point raised by the academic editor and reviewer(s). You should upload this letter as a separate file labeled 'Response to Reviewers'.
- A marked-up copy of your manuscript that highlights changes made to the original version. You should upload this as a separate file labeled 'Revised Manuscript with Track Changes'.
- An unmarked version of your revised paper without tracked changes. You should upload this as a separate file labeled 'Manuscript'.

If you would like to make changes to your financial disclosure, please include your updated statement in your cover letter. Guidelines for resubmitting your figure files are available below the reviewer comments at the end of this letter.

If applicable, we recommend that you deposit your laboratory protocols in protocols.io to enhance the reproducibility of your results. Protocols.io assigns your protocol its own identifier (DOI) so that it can be cited independently in the future. For instructions see: https://journals.plos.org/plosone/s/submission-guidelines#loc-laboratory-protocols. Additionally, PLOS ONE offers an option for publishing peer-reviewed Lab Protocol articles, which describe protocols hosted on protocols.io. Read more information on sharing protocols at https://plos.org/protocols?utm_medium=editorial-email&utm_source=authorletters&utm_campaign=protocols.

As the corresponding author, your ORCID iD is verified in the submission system and will appear in the published article. PLOS supports the use of ORCID, and we encourage all coauthors to register for an ORCID iD and use it as well. Please encourage your coauthors to verify their ORCID iD within the submission system before final acceptance, as unverified ORCID iDs will not appear in the published article. *Only* the individual author can complete the verification step; PLOS staff *cannot* verify ORCID iDs on behalf of authors.

We look forward to receiving your revised manuscript.

Kind regards,

Selvakumar Subbian, Ph.D.

Academic Editor

PLOS One

**Journal Requirements:**

1. When submitting your revision, we need you to address these additional requirements. Please ensure that your manuscript meets PLOS ONE's style requirements, including those for file naming. The PLOS ONE style templates can be found at https://journals.plos.org/plosone/s/file?id=wjVg/PLOSOne_formatting_sample_main_body.pdf and https://journals.plos.org/plosone/s/file?id=ba62/PLOSOne_formatting_sample_title_authors_affiliations.pdf-->2. Your ethics statement should only appear in the Methods section of your manuscript. If your ethics statement is written in any section besides the Methods, please move it to the Methods section and delete it from any other section. Please ensure that your ethics statement is included in your manuscript, as the ethics statement entered into the online submission form will not be published alongside your manuscript.3. Please include captions for your Supporting Information files at the end of your manuscript, and update any in-text citations to match accordingly. Please see our Supporting Information guidelines for more information: http://journals.plos.org/plosone/s/supporting-information.4. Please upload a new copy of Figure S3, as the detail is not clear. Please follow the link for more information:  https://journals.plos.org/plosone/s/figures5. If the reviewer comments include a recommendation to cite specific previously published works, please review and evaluate these publications to determine whether they are relevant and should be cited. There is no requirement to cite these works unless the editor has indicated otherwise.

[Note: HTML markup is below. Please do not edit.]

Reviewers' comments:

Reviewer's Responses to Questions

**Comments to the Author**

1. Is the manuscript technically sound, and do the data support the conclusions?

The manuscript must describe a technically sound piece of scientific research with data that supports the conclusions. Experiments must have been conducted rigorously, with appropriate controls, replication, and sample sizes. The conclusions must be drawn appropriately based on the data presented.

Reviewer #1: Yes

Reviewer #2: Yes

**********

2. Has the statistical analysis been performed appropriately and rigorously?

Reviewer #1: Yes

Reviewer #2: Yes

**********

3. Have the authors made all data underlying the findings in their manuscript fully available?

The PLOS Data policy requires authors to make all data underlying the findings described in their manuscript fully available without restriction, with rare exception (please refer to the Data Availability Statement in the manuscript PDF file). The data should be provided as part of the manuscript or its supporting information, or deposited to a public repository. For example, in addition to summary statistics, the data points behind means, medians and variance measures should be available. If there are restrictions on publicly sharing data—e.g. participant privacy or use of data from a third party—those must be specified.

Reviewer #1: Yes

Reviewer #2: Yes

**********

4. Is the manuscript presented in an intelligible fashion and written in standard English?

PLOS ONE does not copyedit accepted manuscripts, so the language in submitted articles must be clear, correct, and unambiguous. Any typographical or grammatical errors should be corrected at revision, so please note any specific errors here.

Reviewer #1: Yes

Reviewer #2: Yes

**********

5. Review Comments to the Author

Please use the space provided to explain your answers to the questions above. You may also include additional comments for the author, including concerns about dual publication, research ethics, or publication ethics. (Please upload your review as an attachment if it exceeds 20,000 characters)

**Reviewer #1:** The manuscript “Inhaled Nitric Oxide in Fibrotic and Advanced Interstitial Lung Disease: A Systematic Review and Meta-Analysis of Randomized Controlled Trials” is well written but requires revisions to benefit the readers.

1. Inclusion of current treatment modalities across different age groups of ILD

2. The authors could justify how the analysis was performed to include both short (20 min)and long term (weeks) of iNO exposure. The authors could include the effects of the same as well as the advantages and disadvantages of short and long term iNO exposure from a clinician perspective in the discussion.

3. The authors have stated “For MVPA, the certainty of evidence was downgraded for serious inconsistency due to substantial heterogeneity”. Please discuss the possible reasons for the same in previous RCTs.

4. The majority of the population were adults over 60 years and male. Please discuss the same in the context of ILD and iNO therapy. Does the conclusion of no clinical improvement be attributed to the population considered. Is iNO administered to any other age group?

5. Would the author recommend the feasibility of inclusion of iNO in routine therapy – in light of advantages and disadvantages considering no safety issue. What are the drawbacks for iNO considering the routine therapy wherever considered. Why were the same restricted to adults in RCTs.

6. To benefit the reader, the final conclusion could include authors perspective and recommendation on use of iNO for ILD

**Reviewer #2:** GENERAL COMMENTS:

The authors conducted a systematic review of the literature to compare the efficacy and safety of inhaled nitric oxide (iNO) versus placebo in patients with interstitial lung disease (ILD). They identified four randomized controlled trials (RCTs), including one crossover trial, involving 274 patients for analysis. Pooled analyses revealed that iNO likely results in little to no difference in 6-minute walk distance (6MWD) compared with placebo, and may also result in little to no difference in adverse events or moderate-to-vigorous physical activity (MVPA). The authors concluded that iNO did not demonstrate improvements in 6MWD or MVPA. While this report includes valuable information, it raises several concerns.

MAJOR COMMENTS:

1. Rationale of the study

The authors state that previous RCTs have demonstrated increases in MVPA, alongside improvements in dyspnea, quality-of-life measures, and oxygenation. However, a phase-3 trial in patients with fILD found no significant differences in activity level, 6MWD or patient-reported outcomes. They justify their study on the basis that the available evidence regarding iNO in ILD is inconsistent and limited to small-scale trials.

King et al. conducted a phase 2 exploratory randomized controlled trial and found that INOpulse was well tolerated and associated with the maintenance of physical activity and an improvement in symptoms in patients with ILD requiring supplemental oxygen (AnnalsATS, 2021). They then conducted a randomized, double-blind, placebo-controlled trial to validate the benefit of ambulatory iNO in patients with fILD requiring supplemental oxygen, concluding that there was no demonstrable benefit to iNO in patients with the disorder (AnnalsATS, 2024).

Are a further systematic review and meta-analysis necessary?

2. Studies included in review

The authors included four RCTs in this systematic review and meta-analysis. The first RCT was conducted by Nathan et al. and enrolled 41 patients. The second RCT was reported by King et al., although Nathan contributed to the study design, data analysis, and manuscript preparation and revision. The third RCT was performed by Nathan et al. to validate the benefits of ambulatory iNO. Thus, these three RCTs were all conducted by the same group. The fourth study was conducted independently of the others. However, this study only included 44 patients. Is it therefore reasonable to conduct a systematic review and meta-analysis?

3. Baseline patient and study characteristics

According to Table 1, Freidkin et al. administered iNO for 20 minutes once. In contrast, Nathan’s group delivered NO consistently for 8 to 16 weeks (at a daily usage rate of >12 hours). Are the results of the study by Freidkin et al. comparable to those of Nathan’s group?

MINOR COMMENTS:

1. Supplementary Table 5

The study by Freidkin et al. was conducted in Israel rather than the United States.

**********

6. PLOS authors have the option to publish the peer review history of their article (what does this mean?). If published, this will include your full peer review and any attached files.

If you choose “no”, your identity will remain anonymous but your review may still be made public.

**Do you want your identity to be public for this peer review?** For information about this choice, including consent withdrawal, please see our Privacy Policy.

Reviewer #1: **Yes:** Radha Gopalaswamy

Reviewer #2: **Yes:** Toshinori Takada

**********

[NOTE: If reviewer comments were submitted as an attachment file, they will be attached to this email and accessible via the submission site. Please log into your account, locate the manuscript record, and check for the action link "View Attachments". If this link does not appear, there are no attachment files.]

To ensure your figures meet our technical requirements, please review our figure guidelines: https://journals.plos.org/plosone/s/figures

You may also use PLOS’s free figure tool, NAAS, to help you prepare publication quality figures: https://journals.plos.org/plosone/s/figures#loc-tools-for-figure-preparation.

NAAS will assess whether your figures meet our technical requirements by comparing each figure against our figure specifications.

# Author comment: Author response to Decision Letter 1

**DOI:** 10.1371/journal.pone.0351862.r002

25 May 2026

Thank you for the opportunity to revise and resubmit our manuscript. We have carefully revised the manuscript in response to the comments from the Academic Editor and reviewers.

Please find our detailed point-by-point responses in the uploaded file labeled “Response to Reviewers.” We have also uploaded a revised manuscript with tracked changes and a clean version of the revised manuscript.

Attachment Submitted filename: Response to Reviewers.docx

# Peer review: Decision Letter 1

**DOI:** 10.1371/journal.pone.0351862.r003

**Authors:** Selvakumar Subbian, Selvakumar Subbian

3 Jun 2026

Inhaled Nitric Oxide in Fibrotic and Advanced Interstitial Lung Disease: A Systematic Review and Meta-Analysis of Randomized Controlled Trials

PONE-D-26-11568R1

Dear Dr. Nakahara,

We’re pleased to inform you that your manuscript has been judged scientifically suitable for publication and will be formally accepted for publication once it meets all outstanding technical requirements.

Within one week, you’ll receive an e-mail detailing the required amendments. When these have been addressed, you’ll receive a formal acceptance letter and your manuscript will be scheduled for publication.

An invoice will be generated when your article is formally accepted. Please note, if your institution has a publishing partnership with PLOS and your article meets the relevant criteria, all or part of your publication costs will be covered. Please make sure your user information is up-to-date by logging into Editorial Manager at Editorial Manager® and clicking the ‘Update My Information' link at the top of the page. For questions related to billing, please contact billing support.

If your institution or institutions have a press office, please notify them about your upcoming paper to help maximize its impact. If they’ll be preparing press materials, please inform our press team as soon as possible -- no later than 48 hours after receiving the formal acceptance. Your manuscript will remain under strict press embargo until 2 pm Eastern Time on the date of publication. For more information, please contact onepress@plos.org.

Kind regards,

Selvakumar Subbian, Ph.D.

Academic Editor

PLOS One

Additional Editor Comments (optional):

In the revised version, the authors have satisfactorily addressed the concerns raised by the reviewers.

Reviewers' comments:

Reviewer's Responses to Questions

**Comments to the Author**

1. If the authors have adequately addressed your comments raised in a previous round of review and you feel that this manuscript is now acceptable for publication, you may indicate that here to bypass the “Comments to the Author” section, enter your conflict of interest statement in the “Confidential to Editor” section, and submit your "Accept" recommendation.

Reviewer #1: All comments have been addressed

**********

2. Is the manuscript technically sound, and do the data support the conclusions?

The manuscript must describe a technically sound piece of scientific research with data that supports the conclusions. Experiments must have been conducted rigorously, with appropriate controls, replication, and sample sizes. The conclusions must be drawn appropriately based on the data presented.

Reviewer #1: Yes

**********

3. Has the statistical analysis been performed appropriately and rigorously?

Reviewer #1: N/A

**********

4. Have the authors made all data underlying the findings in their manuscript fully available?

The PLOS Data policy requires authors to make all data underlying the findings described in their manuscript fully available without restriction, with rare exception (please refer to the Data Availability Statement in the manuscript PDF file). The data should be provided as part of the manuscript or its supporting information, or deposited to a public repository. For example, in addition to summary statistics, the data points behind means, medians and variance measures should be available. If there are restrictions on publicly sharing data—e.g. participant privacy or use of data from a third party—those must be specified.

Reviewer #1: Yes

**********

5. Is the manuscript presented in an intelligible fashion and written in standard English?

PLOS ONE does not copyedit accepted manuscripts, so the language in submitted articles must be clear, correct, and unambiguous. Any typographical or grammatical errors should be corrected at revision, so please note any specific errors here.

Reviewer #1: Yes

**********

6. Review Comments to the Author

Please use the space provided to explain your answers to the questions above. You may also include additional comments for the author, including concerns about dual publication, research ethics, or publication ethics. (Please upload your review as an attachment if it exceeds 20,000 characters)

Reviewer #1: (No Response)

**********

7. PLOS authors have the option to publish the peer review history of their article (what does this mean?). If published, this will include your full peer review and any attached files.

If you choose “no”, your identity will remain anonymous but your review may still be made public.

**Do you want your identity to be public for this peer review?** For information about this choice, including consent withdrawal, please see our Privacy Policy.

Reviewer #1: **Yes:** Radha Gopalaswamy

**********

# Editor report: Acceptance letter

**DOI:** 10.1371/journal.pone.0351862.r004

**Authors:** Selvakumar Subbian, Selvakumar Subbian

PONE-D-26-11568R1

PLOS One

Dear Dr. Nakahara,

I'm pleased to inform you that your manuscript has been deemed suitable for publication in PLOS One. Congratulations! Your manuscript is now being handed over to our production team.

At this stage, our production department will prepare your paper for publication. This includes ensuring the following:

* All references, tables, and figures are properly cited

* All relevant supporting information is included in the manuscript submission,

* There are no issues that prevent the paper from being properly typeset

You will receive further instructions from the production team, including instructions on how to review your proof when it is ready. Please keep in mind that we are working through a large volume of accepted articles, so please give us a few days to review your paper and let you know the next and final steps.

Lastly, if your institution or institutions have a press office, please let them know about your upcoming paper now to help maximize its impact. If they'll be preparing press materials, please inform our press team within the next 48 hours. Your manuscript will remain under strict press embargo until 2 pm Eastern Time on the date of publication. For more information, please contact onepress@plos.org.

You will receive an invoice from PLOS for your publication fee after your manuscript has reached the completed accept phase. If you receive an email requesting payment before acceptance or for any other service, this may be a phishing scheme. Learn how to identify phishing emails and protect your accounts at https://explore.plos.org/phishing.

If we can help with anything else, please email us at customercare@plos.org.

Thank you for submitting your work to PLOS ONE and supporting open access.

Kind regards,

PLOS ONE Editorial Office Staff

on behalf of

Dr. Selvakumar Subbian

Academic Editor

PLOS One
